# Bone health in Parkinson’s disease: a comprehensive review of bone involvement and its pathophysiological mechanisms

**DOI:** 10.3389/fmed.2025.1737844

**Published:** 2026-01-13

**Authors:** Eric Toussirot, Charline Compagne, Charline Vauchy, Matthieu Bereau

**Affiliations:** 1 Université Marie et Louis Pasteur, Centre d’Investigation Clinique INSERM CIC-1431, CHU de Besançon, Besançon, France; 2 Centre Expert Parkinson, Service de Neurologie électrophysiologie clinique, CHU de Besançon, Besançon, France

**Keywords:** bone mineral density, bone quality, falls, fractures, osteoporosis, Parkinson’s disease

## Abstract

Parkinson’s disease (PD) is a frequent neurodegenerative disorder that combines motor and non-motor features, including impaired balance, gait disturbances, and progressive loss of mobility. Bone involvement is well established, with low bone mass and elevated fracture risk- especially hip fractures- being common findings. Because of impaired balance, gait disturbances, cognitive dysfunction, and autonomic failure, individuals with PD experience a markedly elevated risk of falls. Osteoporosis in PD likely results from a convergence of nutritional deficiencies, vitamin D insufficiency, weight loss with sarcopenia, and progressive muscle weakness. Anti-Parkinson medications such as levodopa may also contribute through hyperhomocysteinemia. In addition, dopamine depletion and chronic inflammation may further disrupt bone remodeling. This review summarizes current evidence on bone mineral density, bone quality, falls, and fractures in PD and discusses the pathophysiological mechanisms underlying this comorbidity.

## 1 Introduction

Parkinson’s disease (PD) is the second most prevalent neurodegenerative disorder after Alzheimer’s disease (1, 2). It manifests with a wide range of motor and non-motor symptoms that substantially burden patients. The key motor manifestations include resting tremor, rigidity, and bradykinesia, often accompanied by disturbances of balance and gait. These features arise from progressive degeneration of dopaminergic neurons in the *substantia nigra pars compacta*, where abnormal accumulation of *α*-synuclein (Lewy bodies) disrupts neuronal survival and function. As dopamine signaling diminishes, corticobasal ganglia circuits become dysfunctional, producing the hallmark motor features. PD is now understood as a complex neuropsychiatric disorder, with non-motor features such as mood and behavioral changes, autonomic dysfunction, and in later stages, cognitive impairment and axial postural abnormalities including scoliosis, camptocormia, and Pisa syndrome (3, 4). These complications, together with nutritional deficits, muscle weakness, and body weight changes, contribute to falls and loss of independence, significantly reducing quality of life for patients and caregivers (5). Accumulating evidence indicates that PD is also complicated by osteoporosis and skeletal fragility (6–9). Compared with individuals without PD, patients show lower bone mineral density (BMD) (10) and an increased risk of falls, leading to higher rates of fragility fractures, especially hip fractures (8, 11). Such fractures carry serious consequences, including disability, hospitalization, and higher mortality (7). This review aims to summarize evidence on bone involvement in PD, focusing on BMD, bone quality, falls, and fractures, and to discuss mechanisms that may underlie these complications.

## 2 Search strategy

A comprehensive literature search was conducted in the Medline database. The search terms included “Parkinson’s disease” combined with “osteoporosis,” “fracture,” “fall risk,” “bone mineral density,” or “bone quality.” The search was restricted to articles published in English. Eligible studies included original research articles, epidemiological studies, systematic reviews, and meta-analyses. Case reports, case series, editorials, conference abstracts, and full-text articles published in languages other than English were excluded. The literature search was performed in June 2025 and covered articles published from 1990 up to the search date.

## 3 Bone mineral density in patients with Parkinson’s disease

Compared with age- and sex-matched controls, individuals with PD consistently exhibit reduced BMD (6, 8, 9), as demonstrated in both cross-sectional and longitudinal studies (12–17). Smaller case–control cohorts have shown lower BMD across multiple skeletal sites, with evidence of progressive decline during follow-up (17). In large prospective cohorts, such as the Study of Osteoporotic Fractures (16), hip BMD was initially lower in PD patients, although differences diminished after adjustment for confounders. Data from the Osteoporotic Fractures in Men study indicated that men with PD experience an accelerated decline in hip BMD relative to non-PD men (age-adjusted mean annualized total hip bone loss: 1.08% vs. 0.36%) (15). Another study found that lower lumbar spine BMD in PD was related to the severity of the disease according to Hoehn and Yahr stage, although this study was limited by the small patient sample size and by the use of dual photon absorptiometry for BMD measurements, rather than the reference method of dual X-ray absorptiometry (DXA) (18). Other studies have suggested that reduced lumbar spine BMD is associated with more advanced disease severity. A large Japanese cohort further linked lower BMD to higher fracture risk, greater disease severity, longer duration, lower body mass index (BMI), and vitamin D deficiency (19). Meta-analyses confirm a consistent pattern: PD is associated with lower BMD at several skeletal sites and an increased prevalence of osteoporosis, though the magnitude of risk may differ by sex. A first meta-analysis reported that PD patients were at a higher risk of osteoporosis than healthy subjects, with an Odd Ratio (OR) of 1.18 [95% CI: 1.09–1.27]. The results showed that PD patients had lower BMD in the hip, lumbar spine and femoral neck than healthy subjects. In addition, the risk of osteoporosis was higher in male patients than in female patients (OR: 2.44 [95% CI: 1.37–4.34] vs. 1.16 [95%CI: 1.07–1.26], respectively) (20). A second meta-analysis reached similar conclusions, finding a higher risk of osteoporosis in PD patients than in healthy controls, with an OR of 2.61 [95% CI: 1.69–4 0.03]. However, unlike the previous study, the risk was higher for female patients. Patients with PD had lower hip, lumbar spine and femoral neck BMD than healthy controls (21). These results were in agreement with a third meta-analysis on BMD in PD patients, which reported lower total body, total hip, total radius, lumbar spine, total femur, femoral neck and hand BMD than in non-PD controls (22) (Table 1). Overall, BMD was lower in patients with Parkinson’s disease compared with the control population, and this reduction was observed at multiple skeletal sites. Whether the decrease in BMD in PD differs by sex remains a matter of debate.

**Table 1:** Meta-analysis on bone mineral density and fracture risk in patients with Parkinson’s disease.

Author Reference year	Outcome	Included studies	Number of studies	Number of patients/controls	Main results
Zhao Bone 2013	Risk of osteoporosis and BMD levels	Comparative studies of patients with PD and healthy controls	15	Risk of osteoporosis: 5 studies with 12,311 PD patients and 134,554 controls BMD: 11 studies with 249–637 PD patients and 550–8,800 controls	Risk of osteoporosis: higher for PD patients (OR 1.18 [95%CI:1.09, 1.27]); higher risk for male patients PD patients have lower hip, lumbar spine and femoral neck BMD than healthy controls.
Thorsney J Neurol Neurosurg Psychiatry 2014	Risk of osteoporosis, fractures and BMD levels	Observational studies with a cohort or case- control design	23	Diagnosis of osteoporosis: 2 studies BMD: 14 studies with 938 and 15,050 controls Fracture risk: 9 studies	Risk of osteoporosis: higher for PD patients (OR 2.61; 95%CI: 1.69–4.03). Male patients had a lower risk of osteoporosis and osteopenia than female patients (OR 0.45; 95% CI 0.29–0.68) PD patients had lower hip, lumbar spine and femoral neck BMD levels compared with healthy controls Risk of fractures: increased in PD patients (OR 2.28; 95% CI 1.83–2.83).
Liu World Neurosurg 2021	Association between PD and BMD	Cohort, case-controlled, or cross-sectional studies.	17	10,289 individuals: 1090 PD and 9,199 non-PD controls.	PD patients: lower total body, total hip, total radius, lumbar spine, total femur, femur neck, right-hand, and left-hand BMD than non-PD controls.
Tan PlosOne 2014	Association between PD and risk of fracture	Prospective cohorts	6	69,387 participants	Risk of fracture: increased in PD compared with control subjects (pooled HR: 2.66, 95% CI: 2.10–3.36).
Hosseinzadeh Acta Neurol Belg 2018	Association between PD and risk of hip fracture	Observational studies	13	564,947 participants	HR overall: 3.13, 95%CI: 2.53–3.87 HR in women: 3.11 95%CI 2.51–3.86 HR in men: 2.60 95%CI 2.19–3.09
Schini Bone 2020	Association between PD and risk of hip and non-vertebral fractures	Observational studies	18	Observational studies	Effect size: risk for hip fractures: 2.40, 95% CI 2.04–2.82 and non-vertebral fractures: 1.80, 95%CI 1.60–2.01 Relative risk for hip fractures higher in men (2.93, 95%CI: 2.05–4.18 than in women: 1.81, 95%CI 1.61–2.04).

BMD, bone mineral density; PD, Parkinson’s disease; OR, odds ratio; RR, relative risk; HR, hazard ratio; CI, confidence interval.

## 4 Bone quality in patients with Parkinson’s disease

Most studies of bone involvement in PD have relied on DXA, which does not capture microarchitectural features of bone. Trabecular Bone Score (TBS), a surrogate marker of bone microarchitecture, has been shown to improve fracture risk prediction in other populations but has only rarely been applied in PD. In smaller clinical studies, results have been mixed: some reported alterations in TBS despite preserved BMD (23), while others found differences more evident when muscle performance was impaired (24): a first study conducted in Ukraine reported TBS evaluation in a series of 38 male patients with PD and 38 without PD. Patients with PD had osteoporosis according to total body BMD measurements, but BMD at the lumbar spine and femoral neck did not differ between the patients and the controls. The TBS assessment showed significantly higher results in the PD group than in the control group (23). Another study from Italy compared 13 patients with PD in the early stages of the disease to 13 age-matched controls. While there was no decrease in BMD among the patients, TBS significantly decreased in the PD group, as determined by the muscle performance assessment using the short physical performance battery (SPPB). Indeed, patients with lower SPPB scores had lower TBS scores despite having normal BMD values (24). These findings suggest that microarchitectural deterioration may occur earlier than measurable BMD loss. Other techniques such as calcaneal ultrasound have also indicated poorer bone quality in PD, with significant differences observed particularly in women (25). Overall, bone quality parameters are altered in PD, as evidenced by trabecular bone score or ultrasound assessments.

## 5 Risk of falls in Parkinson’s disease

Because of balance instability, gait impairment, autonomic dysfunction, and cognitive decline, patients with PD have a markedly increased risk of falling (11). In a prospective cohort of more than 200 patients followed for 8 years, over two-thirds experienced falls, with prevalence rising steadily over time. Factors such as freezing of gait, higher levodopa exposure, and axial motor impairment were identified as predictors (26). Retrospective analyses confirm that around one-third of patients are recurrent fallers, with risk increasing in older individuals, those with more advanced disease, or with prominent rigidity and postural deficits (11). In a large survey, more than half of PD patients reported at least one fall over a two-year period, particularly in the presence of atypical Parkinsonism or dementia (27). Meta-analyses indicate that nearly half of PD patients experience a fall within 3 months, and that a history of recurrent falls (two or more falls in the previous year) is the strongest predictor of future events (28). Patients with PD presenting with postural instability and gait disturbances were more likely to experience falls than those with tremor-dominant symptoms. In a prospective cohort study of 113 patients with PD, the postural instability and gait difficulty subgroup experienced a higher number of falls related to freezing of gait, balance impairment, and falls occurring at home (29). Overall, these studies consistently report a markedly increased risk of falls among patients with PD.

## 6 Risk of fracture in Parkinson’s disease

Multiple retrospective and prospective studies have consistently shown that individuals with PD face a higher burden of fragility fractures compared with age-matched controls. In a population-based study in the USA (Olmsted County, Minnesota), patients with PD were more likely to have a history of fracture than control subjects, with fracture rates of 59 and 44%, respectively, at 10 years. Fractures of the femoral neck accounted for 23% of the fractures in PD patients (30). A retrospective analysis of 200 PD patients and 200 control subjects revealed that fractures were significantly more frequent in the PD group (15% vs. 7.5%). The most common fracture site for PD patients was the femur. Vertebral fractures were also higher in the PD group (31). In long-term population cohorts, fracture incidence in PD exceeded that in non-PD groups, with hip fractures disproportionately represented. Case–control analyses likewise demonstrate that fractures, particularly of the femur and spine, occur significantly more often in PD. The effect of comorbidities on fracture risk was evaluated in the multinational GLOW cohort, which included 52,960 women. This large multinational cohort identified PD as one of the strongest comorbid predictors of fracture risk, even after accounting for other chronic diseases (32). Meta-analysis evidences indicate a roughly two- to threefold increased risk of both hip and non-vertebral fractures, with men appearing especially vulnerable to hip fractures (33–35) (Table 1). Importantly, fractures may occur during the prodromal phase, as patients who later develop PD already show higher fracture rates in the years preceding diagnosis (36). Overall, fracture risk is increased in patients with PD, particularly at the hip, while vertebral fractures are also overrepresented in this population.

## 7 Pathophysiological mechanisms of osteoporosis in PD

**Figure 1:**
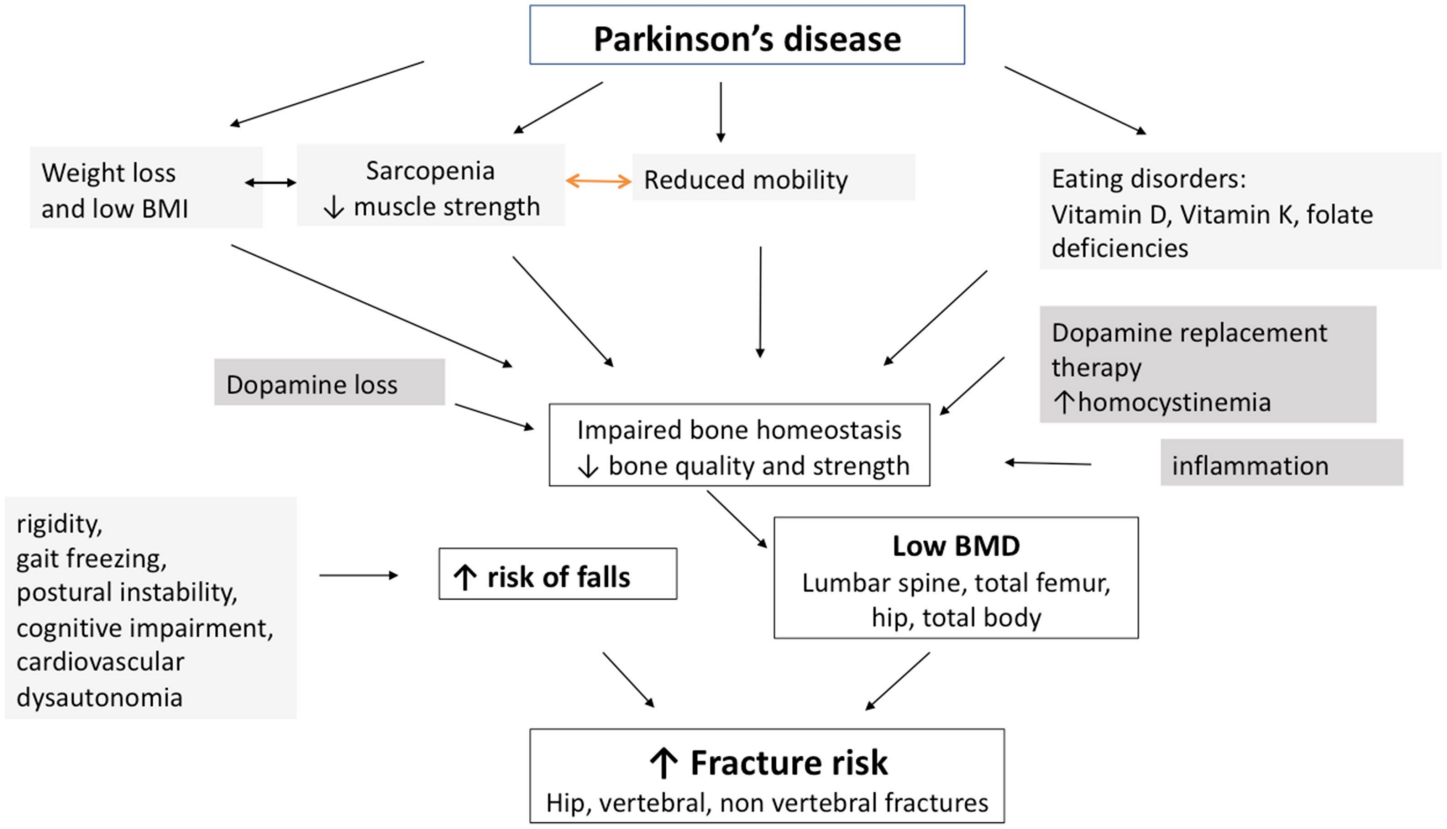
Mechanisms involved in osteoporosis in PD. Patients with PD are characterized by a low BMD resulting from multiple factors that act synergistically: nutritional factors, such as vitamin deficiencies, especially vitamin D; reduced mobility; and changes in body weight and body composition, with weight loss and decreased muscle mass leading to sarcopenia and poor muscle strength. In addition, PD medications may influence bone metabolism, as levodopa treatment induces hyperhomocysteinemia. Presumably, dopamine loss, together with an inflammatory background, contributes to impaired bone remodeling. Bone quality is also altered in PD. The motor symptoms of PD are associated with gait difficulties and postural instability, cognitive decline and dysautonomia, increasing the risk of falls. Ultimately, the combination of low BMD and an increased risk of falls leads to an increased risk of fractures, especially at the hip (PD: Parkinson’s disease; BMD: bone mineral density).

As a systemic neurodegenerative disease involving multiple comorbidities, the mechanisms explaining bone loss and fragility in PD are complex and multifactorial (Figure 1).

### 7.1 Vitamin D and dietary deficiencies

Vitamin D is essential for bone health, and its deficiency has been consistently associated with low bone mass, higher fall risk, and greater fracture incidence (37). Meta-analyses indicate that circulating 1,25 dihydroxy vitamin D concentrations are significantly reduced in PD, and deficiency states are linked to increased disease risk (38). Similar trends are seen when comparing PD with other neurodegenerative disorders such as Alzheimer’s disease (39). In 186 patients with early-stage PD, osteoporosis and osteopenia, as defined by DXA measurements, were commonly observed (12 and 41% respectively). PD patients had lower 1,25 dihydroxy vitamin D levels than 802 controls. Female sex, low body weight and low 1,25 dihydroxy vitamin D levels were associated with bone loss (40). 1,25 dihydroxy vitamin D, the active vitamin D metabolite, can be synthesized in the brain by neurons and microglia, and dopaminergic neurons in the *substantia nigra* express vitamin D receptors. In addition, an association was found between *VDR* gene polymorphisms and PD (41). It has been postulated that chronic inadequate intake of vitamin D could favor a loss of dopaminergic neurons in the brain and thus could contribute to the development of PD. Indeed, 1,25 dihydroxy vitamin D activates VDR for normal cellular function and the *substantia nigra* is dependent on this pathway (42–44). The relationship between vitamin D deficiency and the development of PD was investigated in a prospective cohort study conducted in Finland. This study included 3,173 non-PD subjects who were followed up for 29 years. The results showed that individuals with higher serum 1,25 dihydroxy vitamin D levels had a reduced risk of PD (45). This supports the hypothesis that chronic vitamin D insufficiency may contribute both to neuronal vulnerability and bone fragility. Long-term cohort data suggest that higher 1,25 dihydroxy vitamin D levels reduce the risk of developing PD. Sunlight exposure is the primary source of vitamin D. In patients with PD, sunlight exposure has been shown to improve hypovitaminosis D and BMD, ultimately reducing the risk of hip fracture (46, 47).

Other vitamin deficiencies are also frequent, including folate, B12, and vitamin K, with the latter being positively associated with BMD in advanced PD (48).

### 7.2 Body composition and sarcopenia in Parkinson’s disease

Compared with healthy individuals, patients with PD consistently present with lower BMI, as confirmed by meta-analyses (49, 50). Weight loss may appear early in the disease and typically worsens in advanced stages, particularly when cognitive decline develops (17). Longitudinal studies show that BMI begins to decline within a few years after diagnosis and subsequently decreases more rapidly (51). Nutritional deficits in PD are multifactorial, arising from difficulties with swallowing and feeding, gastrointestinal dysfunction, cognitive impairment, and side effects of PD treatments (6, 8, 9, 52). Body composition is altered in PD with reduced fat mass and lean mass, indicating that weight loss reflects alterations in overall body composition rather than BMI alone (6). Case–control studies confirm lower adiposity measures in PD compared with controls, while muscle mass appears relatively preserved in some cohorts (53). Sarcopenia represents another significant problem: in a large series of 679 male patients with PD from the United Kingdom and Belgium, lean mass and fat mass parameters were measured using DXA. The results showed that 12% of PD patients had sarcopenia according to relative appendicular skeletal muscle mass (RASM < 7.26 kg/m^2^). The analysis showed an association between lean mass (appendicular lean mass), RASM and fat mass with BMD. Men with sarcopenia were more likely to have osteoporosis than patients with normal RASM (54). Frailty frequently overlaps with sarcopenia. In comparative studies, both conditions were far more common in PD than in controls and were associated with disease severity, advanced stage, falls, and institutional care (55). Appetite and energy metabolism changes likely contribute, with reduced caloric intake, higher energy expenditure, and hypothalamic dysfunction all implicated (6, 43). Mechanical loading is a critical driver of bone remodeling, and reduced mobility in PD-manifested as bradykinesia or hypokinesia- may therefore impair bone health. In addition, sarcopenia may also impair bone formation in PD (43). Observational studies link low physical activity and diminished muscle strength with decreased BMD, whereas higher levels of both are associated with greater bone mass (16). In women with PD, impaired gait and lower limb strength have been directly related to reduced hip BMD, illustrating the muscle–bone connection (56). Other investigations confirm pronounced weakness in hip and knee muscles, which likely contributes to bone fragility (57). Notably, muscle impairment can appear even in early disease stages, suggesting a role in the initiation of bone loss (9).

### 7.3 Impact of Parkinson’s disease treatments

Several antiparkinsonian treatments, particularly high-dose levodopa, have been implicated in adverse effects on bone health (58). Large pharmacoepidemiological studies indicate that dopaminergic therapies may increase fracture risk, especially at higher doses of levodopa: this analysis showed that ongoing use of dopaminergic drugs was associated with an increased risk of hip or femur fracture compared to patients who had never taken dopaminergic drugs (OR: 1.76 [95% CI: 1.39–2.22]) (59). Most other drug classes do not appear to significantly influence skeletal outcomes, although high-dose MAO-B inhibitors may elevate hip fracture risk (60). Levodopa therapy is also associated with elevated homocysteine levels (61), and hyperhomocysteinemia has been linked to both lower BMD and higher fracture rates (62). Mechanistic studies suggest that excess homocysteine impairs osteoblast and osteoclast function, thereby disrupting bone remodeling (63, 64).

### 7.4 Effects of dopamine on bone metabolism

Autonomic dysfunction in PD underscores a potential link between nervous system changes and bone fragility, since sympathetic activation can favor bone resorption over formation (43). Animal studies show that disruption of dopaminergic signaling and sympathetic stimulation reduce osteoblast activity and compromise bone remodeling (65). The presence of dopamine receptors on osteoblasts and osteoclasts further supports a direct role for dopamine in bone regulation. *In vitro* and *in vivo* experiments suggest that dopaminergic depletion enhances osteoclast activity while suppressing osteoblast mineralization, ultimately weakening bone integrity (66).

### 7.5 A role for inflammation in Parkinson’s disease bone loss?

Immune dysfunction is increasingly recognized as a contributor to both the onset and progression of PD (67). Epidemiological studies have identified associations between PD and autoimmune disorders, and genetic risk factors for PD overlap with those implicated in autoimmunity (68). Within the brain, neuroinflammation is mediated by activated microglia and elevated levels of pro-inflammatory cytokines including TGFβ, IL-1β, IL-2, IL-6 and TNFα are observed in the brain and cerebrospinal fluid of patients with PD (69–71). Cellular and humoral immune responses are also disturbed in the peripheral blood of patients with PD, with the activation of peripheral monocytes and CD4^+^ T cells with a Th1 and Th17 phenotype, contributing to the production of TNFα and IFNγ (72). Systemic markers such as C-reactive protein are consistently higher in PD and correlate with both motor and non-motor symptoms (73, 74). Since chronic inflammation drives bone loss in autoimmune diseases like rheumatoid arthritis and lupus (75, 76), similar immune-mediated mechanisms may underlie bone fragility in PD.

## 8 Conclusion

Robust evidence indicates that PD impacts bone integrity from early in the disease course. Patients consistently exhibit lower BMD than non-PD individuals, and when combined with frequent falls, this markedly increases hip fracture risk. Skeletal fragility in PD arises from multiple mechanisms, including vitamin D deficiency, body composition changes, sarcopenia, reduced mobility, medication effects such as levodopa-related hyperhomocysteinemia, and systemic inflammation. Nevertheless, osteoporosis screening is not standard practice in PD, and clinical guidelines remain inconsistent. There is a clear need for dedicated recommendations and proactive management strategies to improve bone health outcomes in this vulnerable population.
